# Structured psychiatric care and psychosocial support during placebo participation: association with violent and domestic-violence offending in the ReINVEST trial

**DOI:** 10.3389/fpsyt.2026.1843657

**Published:** 2026-08-12

**Authors:** Emaediong I. Akpanekpo, Lee Knight, Mathew Gullotta, Peter W. Schofield, Tony Butler

**Affiliations:** 1 School of Population Health, University of New South Wales, Sydney, NSW, Australia; 2 School of Medicine and Public Health, The University of Newcastle, Newcastle, NSW, Australia

**Keywords:** domestic violence, forensic psychiatry, placebo trial participation, structured clinical contact, violent offending

## Abstract

**Background:**

Participants in the ReINVEST randomized placebo-controlled trial of sertraline, conducted among men with high trait impulsivity and histories of violent offending, received structured clinical contact throughout the trial, including psychiatric assessments, nursing consultations, crisis support, and referrals to mental health and external services. We estimated the association between placebo trial participation and violent and domestic-violence reoffending, compared with non-participation after baseline and single-blind run-in.

**Methods:**

This prespecified secondary analysis included men from the ReINVEST trial pathway who completed baseline assessment and entered the single-blind run-in phase but did not proceed to randomization, to inform the counterfactual. Violent and domestic-violence offences were identified from linked administrative records over 12- and 24-month follow-up periods. Adjusted differences, targeting the average treatment effect on the treated (ATT), were estimated using g-computation after common-support restriction, with entropy balancing as a second estimator.

**Results:**

Placebo trial participation was associated with lower offending across both outcome domains and follow-up periods. Placebo-standardized mean count differences for violent offending were −0.19 (95% confidence interval [CI] −0.38, −0.04) at 12 months and −0.22 (95% CI −0.51, −0.05) at 24 months. Corresponding differences for domestic-violence offending were −0.37 (95% CI −0.81, −0.14) at 12 months and −0.49 (95% CI −0.92, −0.22) at 24 months. The association was more apparent among men with a documented psychiatric history and, for domestic-violence offending, among those with higher baseline anger, irritability and aggression. Pre-randomization referrals did not explain selection into placebo participation or materially alter the estimates. Post-randomization referrals were observed in both groups, remained more common in the placebo group, and did not account for the observed association.

**Conclusion:**

Placebo participation in this trial involved sustained clinical contact and psychosocial support beyond exposure to inactive medication, and the findings are consistent with a contribution of these non-pharmacological components to lower reoffending. In placebo-controlled trials involving populations with high psychiatric morbidity and limited continuity of coordinated care, the clinical content of placebo participation should be explicitly characterized in trial design and interpretation.

**Clinical trial registration:**

https://www.anzctr.org.au/Trial/Registration/TrialReview.aspx?, identifier ACTRN12613000442707.

## Introduction

Interpersonal violence and domestic and family violence are major contributors to premature mortality, injury-related disability, psychological trauma, and societal cost (1–4). Recidivism rates remain elevated in most countries, and the trajectory from initial conviction to reoffending is accelerated among individuals with complex clinical and social needs (5–8). These characteristics are associated with reduced responsiveness to conventional interventions and with disproportionate contributions to population-level rates of violent crime (9, 10). Despite sustained investment in correctional supervision and rehabilitation programmes, violent and domestic-violence reoffending have remained difficult to reduce (11, 12), generating interest in complementary approaches including pharmacological strategies aimed at the biological mechanisms underlying impulsive aggression (13, 14). These populations are also characterized by high levels of mental health morbidity and poor continuity of mental health care during the transition from custody to the community (15, 16). The intersection of psychiatric morbidity, fragmented service access, and elevated reoffending risk raises the possibility that structured clinical contact, provided within the context of a clinical trial, may represent a meaningful increment in psychiatric care for this population.

One candidate biological pathway involves serotonin regulation. Evidence from neurobiological, pharmacological, and genetic research has linked impaired serotonin signaling to increased impulsive and reactive aggression across a range of populations (17–20). These findings generated the hypothesis that selective serotonin reuptake inhibitors (SSRIs), which increase serotonin availability in the brain, might reduce impulsive aggression among individuals with chronically elevated impulsivity and histories of violent behavior (21–24). However, existing trial evidence has largely come from small studies in psychiatric or forensic settings using laboratory measures or self-reported aggression as proxies for real-world behavior, and it remains unclear whether effects on impulsivity translate into reductions in actual reoffending among community-based justice-involved populations. This evidence gap provided the rationale for the ReINVEST trial (25).

ReINVEST was designed as a pharmacological efficacy trial, but its implementation involved substantial clinical support to facilitate retention and ensure duty of care (26). Participants had sustained contact with psychiatrists, mental health nurses, and psychologists, and clinician-participant interactions extended beyond protocol to include case management, crisis support, service referral, and liaison with courts and other agencies (27). In the conventional logic of a placebo-controlled trial, the placebo arm is intended to estimate outcomes in the absence of pharmacological effect. However, in a trial where both arms received intensive and sustained clinical contact that extended beyond protocol, the placebo condition may not be equivalent to the absence of clinical input. Placebo participation plausibly represented exposure to a structured package of psychiatric monitoring, clinical support, referral, and advocacy that, for many participants, exceeded the level of coordinated mental health care they would otherwise have received. Post-trial linkage of trial records to the state reoffending database showed that offending was higher among men who entered the run-in but did not proceed to randomization than among men randomized to placebo or sertraline (27); this finding was not protected by randomization. The difference could reflect the clinical and psychosocial content of trial participation, pre-existing differences between men who did and did not progress to randomization, or both, and the earlier regression-adjusted results published in the parent trial could not distinguish these.

The hypothesis that the clinical content of trial participation may influence offending draws on two established perspectives. Common factors models of therapeutic change attribute a large share of improvement across treatment modalities to the relational elements of clinical encounters, including alliance, attention, and expectancy (28, 29). Routine activity theory locates offending opportunities in everyday activity patterns and identifies unstructured time without capable guardianship as a context for offending ( (30, 31). Scheduled assessments, weekly telephone check-ins, and engagement with referral services supply both relational contact and structured time, and both perspectives predict lower offending with sustained clinical contact.

This study was designed to address these gaps and was limited to: 1) placebo participants and; 2) participants who completed the baseline assessment and entered the single-blind run-in phase but did not proceed to randomization. The primary aim was to estimate the marginal difference in subsequent violent and domestic-violence reoffending associated with entry into the randomized trial structure under placebo, compared to not entering the randomized phase after baseline assessment and entry into the run-in phase. The second aim was to characterize the scope and frequency of clinician-delivered psychiatric and psychosocial supports recorded in the trial’s clinical management system. The third aim was to assess whether the estimated placebo participation effect varied across selected baseline clinical and criminal-history characteristics. This was motivated by evidence that psychiatric history, anger-related constructs, and criminal-history severity moderate treatment response in forensic populations (32–37). The fourth aim was to examine whether external referrals recorded before randomization explained selection into placebo participation or materially altered the estimated participation effect, and whether external referrals recorded after randomization accounted for any part of the estimated association. External referral was a documented component of trial participation, so referrals before randomization could confound the participation contrast and referrals after randomization could lie on the pathway between participation and offending.

Three directional hypotheses guided the analyses. First, placebo participation would be associated with lower violent and domestic-violence reoffending than non-participation at both follow-up horizons. Second, the association would be larger among men with a documented psychiatric history and among those with higher baseline anger-related symptoms. Third, external referrals recorded before randomization would not explain selection into placebo participation, and external referrals recorded after randomization would not fully account for the association, because the core of the participation exposure was the structured clinical contact delivered by the trial team.

## Methods

### Study design and data sources

This prespecified secondary analysis utilised data from the ReINVEST trial, a randomized, double-blind, placebo-controlled trial of sertraline in highly impulsive adult men with histories of violent offending. Trial records were linked to the New South Wales (NSW) Reoffending Database (ROD) to ascertain criminal justice outcomes over 12- and 24-month follow-up periods.

### Trial registration and ethics

The trial protocol has been published previously (25), and the primary results are reported elsewhere (27). The trial was registered with the Australian New Zealand Clinical Trials Registry (ACTRN12613000442707) and received ethical approval from the University of New South Wales (UNSW) Human Research Ethics Committee (HC11390, HC17771), the Aboriginal Health and Medical Research Council (822/11), Corrective Services NSW (09/26576), and NSW Justice Health and Forensic Mental Health Network (G8/14).

### Participants and trial procedures

Eligible participants were adult males aged 18 years or older who were identified through psychiatric assessment as having high impulsivity, defined by a Barratt Impulsiveness Scale total score of at least 70, and a documented history of at least two violent offences. The Barratt Impulsiveness Scale threshold of 70 was used to identify participants with elevated trait impulsivity and was close to the published threshold for high impulsiveness on the BIS-11 (38). The requirement for prior convictions for two or more violent offences identified men with a documented pattern of violent offending rather than a single recorded incident, consistent with the trial’s focus on repeat violent offenders and violent reoffending (25). Participants were recruited through referrals from courts, community corrections agencies, legal representatives, and self-referral. Exclusion criteria included inability to provide informed consent, major mental illness or clinically significant comorbidities, and current use of antipsychotic, anticonvulsant, or serotonergic medications.

Following screening and eligibility assessment, participants who met ongoing eligibility criteria entered a run-in phase during which all participants received open-label sertraline to assess tolerability and willingness to adhere to the follow-up schedule. Individuals who completed the run-in phase and met ongoing eligibility criteria were randomized to continue sertraline or transition to an identical placebo following down-titration. Participants attended protocol visits at baseline, at week two, and then every four weeks until approximately week 50. Clinical consultations were conducted by psychiatrists, mental health nurses, and psychologists throughout the randomized phase.

### Clinical team model and nature of trial participation

The clinical team consisted of mental health nurses and psychologists, supported by psychiatrists, who collectively provided psychiatric assessments and ongoing eligibility screenings, weekly telephone check-in calls, monthly in-person medication reviews, and access to a 24-hour crisis hotline. This structured clinical contact occurred on a regular, predictable schedule and represented sustained engagement with the health and support system throughout the duration of the trial. In addition to protocol-specified tasks, clinicians performed case management activities beyond the formal trial protocol, including advocacy and liaison with external services, referrals to community supports, crisis support extending to court and service attendances, as well as risk escalation and trauma-informed responses. This broader clinical-contact package is relevant to interpretation of the placebo participation effect because it constitutes a plausible non-pharmacologic component of placebo participation in this trial.

### Analytic groups and exposure definition

The placebo group included all participants randomized to placebo as part of the double-blind phase. The counterfactual was informed by men who completed baseline assessment and entered the single-blind run-in phase but did not proceed to randomization (hereafter, the comparison group). These men had entered the same pre-randomization trial pathway but were not exposed to the subsequent structured clinical contact and repeated follow-up visits that characterized participation in the randomized phase. Among the 166 men who entered the run-in but did not proceed to randomization, 28 were excluded after the run-in (17 mental health-related, 6 medically unfit, and 5 other), 133 dropped out after the run-in (75 lost contact, 26 lost interest, 17 medication-related, 12 offence-related, and 3 other), and 5 had no reported reason. A sensitivity analysis addressed the possibility that these pathways differ in their relation to subsequent offending risk by excluding men who left the run-in on medical or psychiatric grounds (**Supplementary Table 11**).

### Estimand and causal assumptions

The study used a causal inference approach based on potential outcomes (39). The primary target of inference was the expected difference in offending that would have been observed among placebo participants had they not entered the randomized phase; that is, what would have happened to the same men under non-participation, with all other factors held constant. This quantity was chosen because the policy-relevant question concerns the impact of trial participation on those who actually enrolled, rather than an average across all men who might have been eligible. Estimation of the ATT is also confined to the covariate distribution of the placebo group, which avoids extrapolation to covariate profiles observed only among men who did not progress to randomization. This target corresponds to the average treatment effect on the treated (ATT), formally E[Y(1) − Y(0) | A = 1], where Y(1) and Y(0) denote the potential offending outcomes under placebo participation and non-participation and A denotes participation status, averaged over the covariate distribution of the placebo group. Valid estimation required three assumptions (40, 41): (1) that participation status was independent of unmeasured confounders given the observed baseline covariates (conditional exchangeability); (2) that all participants had a non-zero probability of being in either group given their covariates (positivity); and (3) that the defined exposure corresponded to a well-specified intervention (consistency). Placebo participation included scheduled psychiatric assessment, nursing consultation, crisis support, case management, and external referral, and the intensity of contact varied across participants. The exposure was defined as entry into the randomized phase under placebo, and the estimand is interpreted as the average effect of participation as delivered in this trial, averaged over the versions of participation that occurred, rather than the effect of any single component delivered at a fixed intensity.

### Outcomes and follow-up

All offending outcomes were ascertained by probabilistic linkage of trial records to the ROD, which captures proven criminal offences from court and incarceration records. Two offending outcome domains were assessed: violent offending and domestic-violence offending. Violent offences were classified using Australian and New Zealand Standard Offence Classification categories 01 to 06, which includes homicide and related offences, acts intended to cause injury, sexual assault and related offences, dangerous or negligent acts endangering persons, abduction and harassment, and robbery and extortion (42). Domestic-violence offences were identified using domestic-violence offence flags within the ROD. Outcomes were analyzed over 12-month and 24-month follow-up horizons from the screening date. The outcome for each horizon was the number of proven offence events occurring during the relevant follow-up window, with multiple offences recorded on the same date treated as a single event and classified according to the most serious offence recorded on that date. Consistent with previous research (5, 7), this approach avoids counting multiple charges from the same incident as separate offending occasions and aligns the outcome with distinct offending events. Person-time at risk in the community during each window was incorporated as a log-transformed offset in all outcome models so that estimated differences reflect offending conditional on time at risk rather than crude differences in event accumulation (43).

### Covariates

Adjustment covariates for the primary participation analyses were selected *a priori* as potential confounders of the participation-offending association. The baseline covariate set included Indigenous status, history of juvenile detention, any prior offending, prior violent offending, prior domestic-violence offending, psychiatric history, baseline depressive symptom severity, baseline anger and irritability, baseline psychological distress, and baseline self-reported physical and mental health functioning. Depressive symptom severity was measured by the Beck Depression Inventory, anger and irritability by the Anger, Irritability and Assault Questionnaire, psychological distress by the Kessler Psychological Distress Scale (K10), and physical and mental health functioning by the 12-Item Short Form Health Survey, yielding Physical and Mental Component Scores (44–48). Each covariate was selected as a potential common cause of both participation status and reoffending. Indigenous status, juvenile detention history, and the prior-offending measures index criminogenic risk that predicts both continued engagement with the trial and subsequent offending. Psychiatric history and the baseline clinical scores index clinical severity and need, which influence both willingness and capacity to remain in the trial and the risk of reoffending. Covariates were measured at or before baseline and therefore precede the participation exposure, so none can act as a mediator or collider on the participation-offending pathway. Where analyses specifically examined the role of pre-randomization external referral history, this set was supplemented by indicators of prior court assistance/support, prior medical services other than mental health services, prior mental health services, prior legal services, and any prior external referral.

### Statistical analysis

We estimated the placebo-standardized marginal difference in offending between placebo trial participation and non-participation. The primary analysis used common-support restriction with g-computation. G-computation is a standardization method that uses a fitted outcome model to predict each participant’s offending count under participation and under non-participation, holding baseline characteristics fixed, and then averages the difference across participants (49, 50). Entropy balancing is a reweighting method that calibrates weights for the comparison group so that its weighted baseline characteristics match those of the placebo group, after which the same outcome model is fitted (51). The placebo-standardized mean count difference is the average difference in predicted offence counts between participation and non-participation, standardized to the baseline characteristics of placebo participants. Covariate balance before and after common-support restriction and entropy balancing was assessed using standardized mean differences (**Table 1**).

**Table 1:** Baseline characteristics by analytic group, whole sample and common-support sample.

Variable		Whole sample (N = 434)				Common support (N = 428)			
	Statistic	Placebo (n = 285)	Entered run-in, not randomized (n = 149)	SMD (pre)	SMD (EB)	Placebo (n = 285)	Entered run-in, not randomized (n = 143)	SMD (pre)	SMD (EB)
Sociodemographic									
Age (years)	Mean (SD)	32.13 (9.86)	30.58 (9.92)	0.16	−0.05	32.13 (9.86)	30.87 (9.95)	0.13	0.00
Indigenous	N (%)	78 (27.4%)	50 (33.6%)	−0.14	0.00	78 (27.4%)	45 (31.5%)	−0.09	0.00
Criminal History									
Prior juvenile detention	N (%)	59 (20.7%)	52 (34.9%)	−0.32	0.00	59 (20.7%)	47 (32.9%)	−0.24	0.00
Any prior offence	Mean (SD)	10.86 (8.51)	14.33 (11.72)	−0.34	0.00	10.86 (8.51)	13.12 (10.16)	−0.28	0.00
Prior violent offence	Mean (SD)	2.58 (2.27)	2.54 (2.03)	0.00	0.00	2.58 (2.27)	2.54 (2.03)	0.00	0.00
Prior DV offence	Mean (SD)	2.89 (3.09)	2.36 (2.52)	0.19	0.00	2.89 (3.09)	2.41 (2.55)	0.17	0.00
Baseline Clinical Measures									
Psychiatric history	N (%)	228 (80.0%)	124 (83.2%)	−0.08	0.00	228 (80.0%)	118 (82.5%)	−0.07	0.00
BDI score	Mean (SD)	11.33 (8.70)	10.74 (8.11)	0.07	0.02	11.33 (8.70)	10.83 (8.04)	0.06	0.02
AIAQ total	Mean (SD)	70.97 (23.30)	74.08 (21.74)	−0.14	0.00	70.97 (23.30)	73.48 (21.80)	−0.11	0.00
K10 score	Mean (SD)	15.23 (9.05)	15.17 (7.78)	0.00	0.00	15.23 (9.05)	15.40 (7.75)	−0.07	0.01
SF-12 PCS	Mean (SD)	53.11 (7.19)	53.59 (7.55)	0.00	0.00	53.11 (7.19)	53.48 (7.63)	0.00	0.00
SF-12 MCS	Mean (SD)	40.77 (12.45)	39.42 (12.16)	0.11	0.00	40.77 (12.45)	39.36 (12.22)	0.12	−0.02

N (%) for binary variables; Mean (SD) for continuous variables. SMD (pre), unadjusted standardized mean difference; SMD (EB), post-weighting SMD after entropy balancing. BDI, Beck Depression Inventory; AIAQ, Anger, Irritability and Aggression Questionnaire; K10, Kessler Psychological Distress Scale; SF-12 PCS/MCS, Short Form-12 Physical/Mental Component Scores; DV, domestic violence; ESS, effective sample size. Common support defined by minimum and maximum propensity scores among placebo participants. Entropy balancing was applied to the common-support sample, giving an effective sample size of 111.6 and a maximum weight of 5.40.

A logistic regression model was fitted for the probability of belonging to the placebo group rather than the comparison group using the baseline covariate set. The analytic sample was then restricted to participants whose estimated propensity score lay within the range observed among placebo participants, to confine inference to the region of shared covariate support. The distributions of estimated propensity scores in the placebo and comparison groups were also inspected graphically to assess positivity (**Supplementary Figure 1**). Within this common-support sample, offending counts were modelled using Poisson regression with a participation indicator, the baseline covariates, and a log-transformed person-time offset. The Poisson model served as a working model for the conditional mean. The target quantity, a marginal mean count difference, depends on correct specification of the conditional mean rather than on the Poisson variance assumption, and confidence intervals were obtained by nonparametric bootstrap rather than from model-based standard errors, so inference does not rely on the equidispersion assumption. Overdispersion was assessed using Pearson dispersion ratios in the common-support outcome models, and a negative binomial specification was fitted as an additional sensitivity analysis. G-computation was used to generate predicted offending counts for each placebo participant under two scenarios: placebo participation as observed and counterfactual non-participation, with baseline covariates held at their observed values. The participation effect was estimated as the average difference between these two sets of predictions across placebo participants. A sensitivity analysis used entropy balancing to target the same estimand while retaining the full analytic sample (49, 52, 53).

Effect-modification analyses were conducted at 12 months post-randomization. Continuous modifiers included prior violent offence count, prior domestic-violence offence count, depressive symptom severity, anger and irritability, psychological distress, and self-reported mental health functioning. Each was measured at baseline and modelled using a natural cubic spline, with interaction terms used to assess whether the participation effect varied across the modifier distribution. Each spline used three degrees of freedom, with knots placed at the 25th, 50th, and 75th percentiles of the modifier distribution in the combined analytic sample. This specification was fixed before examining modifier-by-outcome associations. Participation effects were estimated at the 25th, 50th, and 75th percentiles of each continuous modifier, and the higher-versus-lower contrast was defined as the difference between the effects estimated at the 75th and 25th percentiles. Psychiatric history was examined separately as a binary modifier. Joint Wald tests were used for interaction terms, and percentile confidence intervals were obtained by nonparametric bootstrap.

External referral activity was compared between placebo participants and non-randomized participants, with referral categories treated as non-mutually exclusive. Referrals recorded before randomization were examined as potential confounders by: (1) modelling placebo participation as a function of the pre-randomization referral variables and (2) repeating the main participation analyses after adding these measures to the adjustment set. Referrals recorded after randomization were examined separately as candidate intermediate variables that might account for part of the association between placebo participation and later offending. Indirect, direct, and total components were estimated using single-mediator interventional analyses under the same common-support framework as the main analyses.

Uncertainty was quantified using nonparametric bootstrap resampling at the participant level with replacement, with 1,000 replications per analysis. In each bootstrap replicate, the full estimation procedure was repeated from propensity score estimation through outcome modelling and g-computation. Percentile confidence intervals were derived from the empirical bootstrap distribution. Missing baseline covariate data were handled using multiple imputation by chained equations (20 imputations) under the missing-at-random assumption. The imputation model included all baseline covariates, the exposure, the outcome count, and log time at risk. Within each imputed dataset, the common-support restriction and entropy balancing were re-applied, and estimates were pooled across imputations using Rubin rules. As a supplementary sensitivity analysis for unmeasured confounding, E-values were calculated from placebo-standardized rate ratios derived from the same g-computation predictions used for the ATT. Protective rate ratios were inverted before applying the E-value formula, and confidence-limit E-values used the bootstrap confidence limit closest to the null. All analyses were conducted in Python (version 3.11), using pandas and NumPy for data management, scikit-learn for propensity score estimation, statsmodels for generalized linear models with offset terms and frequency weights, SciPy for entropy-balancing optimization, and joblib for parallelized bootstrapping.

## Results

### Analytic sample and baseline balance

**Figure 1:**
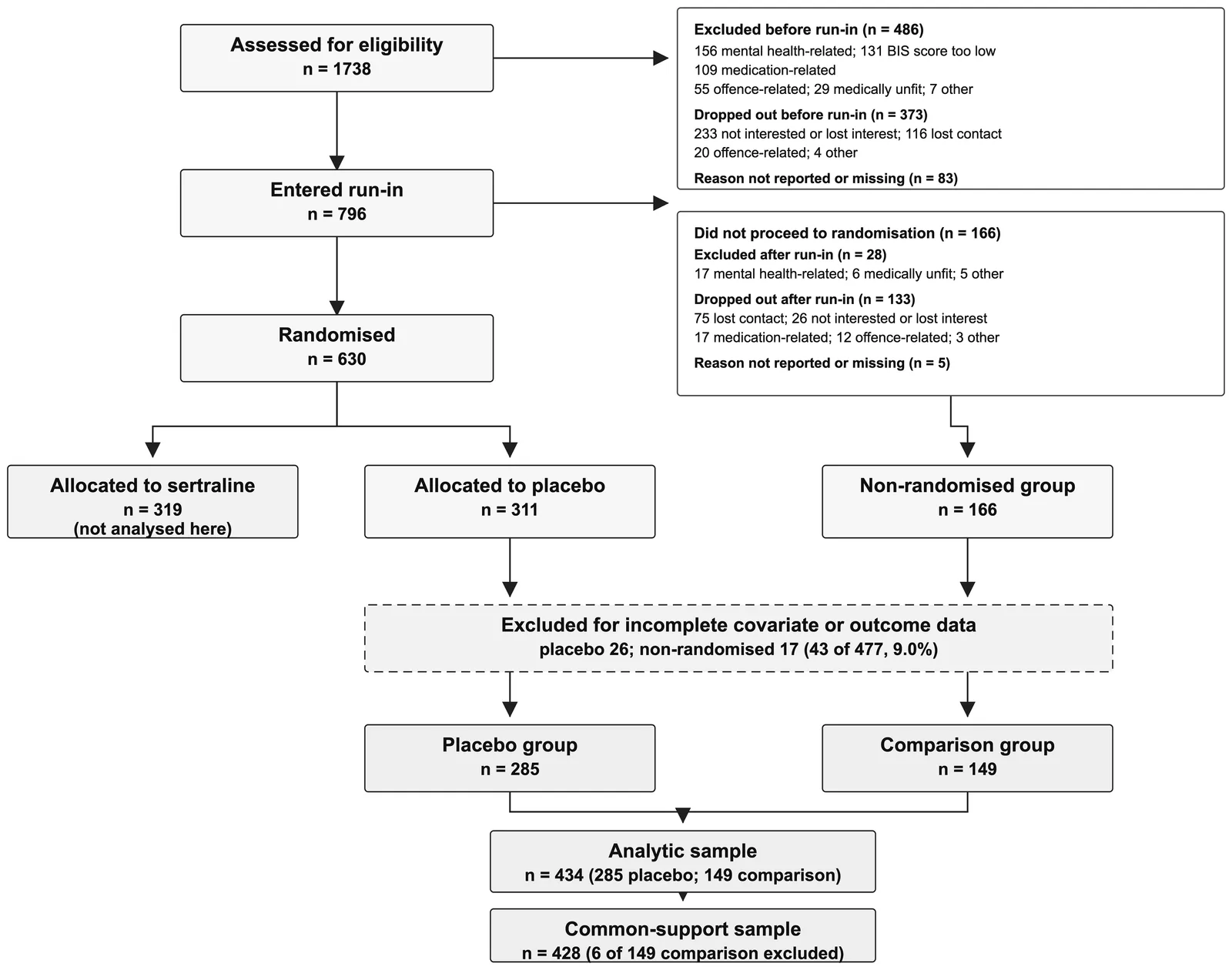
Flowchart of the study population.

The analytic sample comprised 434 men with complete covariate and outcome data, including 285 in the placebo group and 149 in the comparison group (**Figure 1**). Restriction to the common-support region retained 428 participants (all 285 placebo and 143 of 149 comparison-group participants). Entropy balancing reduced measured baseline differences to negligible levels in both the whole sample and the common-support sample (**Table 1**).

### Association of placebo trial participation with reoffending

Placebo trial participation was associated with fewer violent and domestic-violence offences than non-participation under both estimators (**Table 2**). Observed rates in the analytic sample were 31.2 and 54.5 violent offences per 100 person-years in the community at 12 months in the placebo and comparison groups, and 27.7 and 44.0 at 24 months. Corresponding rates for domestic-violence offending were 37.9 and 96.9 at 12 months and 30.7 and 67.9 at 24 months (**Supplementary Table 14**). For violent offending, the placebo-standardized mean count difference was −0.19 (95% confidence interval [CI] −0.38 to −0.04) at 12 months and −0.22 (−0.51 to −0.05) at 24 months under common-support restriction. Estimates under entropy balancing were closely aligned. Differences for domestic-violence offending were larger in absolute magnitude: −0.37 (95% CI −0.81 to −0.14) at 12 months and −0.49 (−0.92 to −0.22) at 24 months under common support (**Table 2**). Multiple imputation by chained equations addressing missing baseline covariate data produced estimates consistent with the complete-case primary analysis across all outcomes and horizons (**Supplementary Table 10**). Furthermore, excluding men who left the run-in on medical or psychiatric grounds produced consistent estimates (**Supplementary Table 11**). Pearson dispersion ratios in the common-support outcome models were 1.40 and 1.45 for violent offending at 12 and 24 months, and 2.04 and 2.13 for domestic-violence offending, and negative binomial estimates were consistent with the primary estimates: −0.16 (95% CI −0.37 to −0.02) and −0.23 (−0.47 to −0.03) for violent offending at 12 and 24 months, and −0.37 (−0.90 to −0.12) and −0.51 (−1.02 to −0.20) for domestic-violence offending. Across the imputation, exclusion, outcome-model, and second-estimator analyses, the direction and approximate magnitude of the association were preserved.

**Table 2:** Placebo-standardized mean count differences for violent and domestic-violence reoffending (N = 428).

Outcome	Follow-up period	Common-support effect (95% CI)	Entropy-balanced effect (95% CI)
Violent offending	12 months	−0.19 (−0.38, −0.04)	−0.20 (−0.40, −0.02)
	24 months	−0.22 (−0.51, −0.05)	−0.23 (−0.50, −0.02)
DV offending	12 months	−0.37 (−0.81, −0.14)	−0.37 (−0.70, −0.09)
	24 months	−0.49 (−0.92, −0.22)	−0.51 (−0.90, −0.17)

In supplementary unmeasured-confounding sensitivity analyses, placebo-standardized rate ratios derived from the same g-computation predictions were below 1.00 across all outcomes and estimators (**Supplementary Table 12**). For violent offending, common-support rate ratios were 0.63 at 12 months and 0.71 at 24 months, with E-values of 2.57 and 2.15. The E-values for the confidence limits closest to the null were 1.19 and 1.24. For domestic-violence offending, common-support rate ratios were 0.50 at 12 months and 0.55 at 24 months, with E-values of 3.38 and 3.01, and confidence-limit E-values of 1.93 and 1.91. Entropy-balanced estimates gave similar point E-values. The 24-month violent-offending confidence-limit E-value was close to the null, reflecting a rate-ratio confidence interval that approached 1.00.

### Effect-modification analyses

At 12 months, the estimated participation effect was larger among men with a documented psychiatric history than among those without, for both violent offending (−0.29 [95% CI −0.53 to −0.03] vs −0.13 [−0.56 to 0.14]) and domestic-violence offending (−0.79 [−1.39 to −0.15] vs −0.08 [−0.73 to 0.16]). For domestic-violence offending, the effect was also larger at the 75th percentile of baseline anger, irritability, and aggression (−0.66 [−1.19 to −0.12]) than at the 25th percentile (−0.34 [−0.99 to 0.08]). The higher-versus-lower contrasts were imprecise in all cases, and the interaction tests did not provide clear evidence of heterogeneity (**Table 3**). The subgroup estimates are exploratory and do not establish differential effects.

**Table 3:** Placebo participation effects on 12-month offending outcomes by baseline characteristic level.

Outcome	Modifier	Level	Common-support effect (95% CI)	High vs. low contrast (95% CI)	Interaction p-value
Violent offending	Psychiatric history	No history	−0.13 (−0.56, 0.14)	Reference	0.400
		Any history	−0.29 (−0.53, −0.03)	−0.16 (−0.51, 0.32)	
Domestic-violence offending	Anger, Irritability and Aggression Score	Low (P25)	−0.34 (−0.99, 0.08)	Reference	1.000
		High (P75)	−0.66 (−1.19, −0.12)	−0.32 (−1.08, 0.43)	
	Psychiatric history	No history	−0.08 (−0.73, 0.16)	Reference	0.082
		Any history	−0.79 (−1.39, −0.15)	−0.71 (−1.33, 0.30)	

12-month placebo-standardized mean count differences (common-support g-computation). Negative values indicate fewer offences under placebo. Continuous modifiers evaluated at the 25th and 75th percentiles using natural cubic splines. p-values are joint Wald tests for modifier-by-participation interaction.

### Referrals to external services

Among participants with structured clinician-contact data, 179 placebo participants generated 524 referral events and 81 non-randomized participants generated 180 referral events (**Table 4**). Court assistance or support was the most frequent category in both groups, accounting for 53.6% of referral events in the placebo group and 59.4% in the non-randomized group. Medical services accounted for 21.4% and 19.4%, mental health services for 13.0% and 13.3%, and legal services for 7.4% and 4.4%, respectively (**Table 4**).

**Table 4:** Referrals to external services recorded by the ReINVEST clinical team.

Referral category	Placebo events (n=524)	Placebo participants (n=179)	Entered run-in, not randomized events (n=180)	Entered run-in, not randomized participants (n=81)
Court assistance/support	281 (53.6%)	137 (76.5%)	107 (59.4%)	61 (75.3%)
Medical services	112 (21.4%)	58 (32.4%)	35 (19.4%)	21 (25.9%)
Mental health services	68 (13.0%)	37 (20.7%)	24 (13.3%)	20 (24.7%)
Legal services	39 (7.4%)	24 (13.4%)	8 (4.4%)	7 (8.6%)
Police	5 (1.0%)	5 (2.8%)	2 (1.1%)	2 (2.5%)
Substance abuse/addiction	11 (2.1%)	5 (2.8%)	0 (0%)	0 (0%)
Charity organisations	3 (0.6%)	1 (0.6%)	1 (0.6%)	1 (1.2%)
Local community services	1 (0.2%)	1 (0.6%)	2 (1.1%)	2 (2.5%)
Disability services	1 (0.2%)	1 (0.6%)	0 (0%)	0 (0%)
Dept. Communities & Justice	2 (0.4%)	1 (0.6%)	0 (0%)	0 (0%)
Employment assistance	0 (0%)	0 (0%)	1 (0.6%)	1 (1.2%)
Victim support services	1 (0.2%)	1 (0.6%)	0 (0%)	0 (0%)

When referrals were examined by timing relative to randomization, external referrals before randomization were more common in the comparison group across the main service categories (**Supplementary Table 1**). After randomization, referrals were recorded in both groups but were more common in placebo participants: court assistance or support for 35.0% versus 16.9%, medical services for 15.2% versus 6.0%, mental health services for 8.1% versus 3.6%, and legal services for 5.5% versus 1.8% (**Supplementary Table 1**).

Pre-randomization external referrals were examined as potential confounders of the participation-offending association. Prior court assistance or support was associated with a lower likelihood of later placebo participation (risk ratio 0.73, 95% CI 0.59 to 0.91), as was any prior external referral (0.73, 0.60 to 0.89) (**Supplementary Table 2**). However, additional adjustment for these variables produced little change in the estimated participation effects (**Supplementary Table 3**).

Post-randomization external referrals were examined as candidate intermediate variables. Indirect components were small and their confidence intervals included zero across all referral categories and both estimators. For domestic-violence offending at 12 months, the total effect was −0.41 (95% CI −0.70 to −0.12) under common support with court assistance as the intermediate variable, and the corresponding indirect component was −0.00 (−0.04 to 0.04) (**Supplementary Table 6**). Results were similar across other referral categories and follow-up periods (**Supplementary Tables 4**-**7**). Post-randomization external referrals did not account for the observed association.

## Discussion

In this prespecified secondary analysis of the ReINVEST clinical trial, placebo participation was associated with fewer subsequent violent and domestic-violence offences than the counterfactual of non-participation. Estimated mean count differences were negative at 12 and 24 months for both outcome domains, with the largest absolute differences observed for domestic-violence offending. The referral profile included mental health services, medical care, and court assistance, consistent with a level of psychiatric care and psychosocial support that extended beyond the procedural requirements of a pharmacological trial. The estimates represent the average difference in offending associated with the full package of placebo participation, weighted to reflect the baseline characteristics of men who were actually randomized to placebo. Additional analyses of external referral activity did not materially alter this interpretation. Adjustment for referrals recorded before randomization produced little change in the estimated participation effects, and referrals recorded after randomization did not account for the association. These findings suggest that the observed association was not readily explained by differences in external service engagement alone and support interpretation of placebo participation as a broader clinical and psychosocial exposure.

The estimated participation effect was more clearly apparent among men with a documented psychiatric history and, for domestic-violence offending, at higher levels of baseline anger and irritability. These analyses were exploratory, the interaction tests did not provide clear evidence of heterogeneity, and the higher-versus-lower comparisons were imprecise. However, the direction of the findings is clinically coherent and aligns with previous studies. In forensic psychiatric populations, anger-related constructs predict aggressive behavior, and anger-control interventions are recognized treatment targets in the management of aggression, including in forensic psychiatric settings (54, 55). Anger and hostility have also been identified as relevant psychological factors in intimate partner violence perpetration (56). Psychiatric trial literature suggests that placebo-arm improvement is shaped in part by treatment context, including expectancy, therapeutic alliance, and other trial features such as visit structure and clinician contact (57–59). It is plausible that men with psychiatric history and elevated anger-related symptoms were more likely to experience the trial’s structured monitoring, crisis support, and referral processes as a clinically meaningful increment in care. The present design cannot determine whether this trend reflects greater responsiveness to structured clinical contact, greater unmet need at baseline, or residual differences not fully captured by measured covariates, and these findings should therefore be interpreted as hypothesis-generating rather than confirmatory. These mechanisms, including therapeutic alliance, expectancy, and the structure of clinical encounters, are theoretically oriented interpretations drawn from the broader placebo and psychotherapy literature. They were not directly tested in this study, and the present design cannot determine which components of the structured clinical contact were responsible for the observed association.

The present findings are consistent with a broader literature documenting that structured, intensive contact between justice-involved individuals and a consistent care or support team may itself be associated with lower reoffending trajectories (60, 61). Conceptually, this aligns with the common factors model of therapeutic change, which posits that relational and contextual elements of clinical encounters, including the therapeutic alliance, empathic attention, and the expectation of benefit, account for a substantial portion of improvement across therapeutic modalities, independent of the specific treatment delivered (28, 29). The routine activities perspective offers a complementary interpretation. Offending opportunities are shaped by everyday activity patterns, including the convergence of likely offenders, suitable targets, and limited guardianship (30). Structured clinical appointments may alter these routines by replacing some unstructured time with scheduled contact with adults or service providers, a mechanism consistent with Osgood and colleagues’ account of unstructured socializing with peers in the absence of authority figures as an opportunity context for deviance (31). Durable reductions in violent and domestic-violence recidivism have proved difficult to achieve across evaluated programmes. Meta-analytic evidence from randomized trials of psychological interventions for incarcerated populations has shown modest aggregate effects that attenuate when small-study bias is accounted for (12); meta-analyses of batterer intervention programmes have reported weak effects on proven reoffending (62, 63); and a more recent meta-analysis of specialized offence-focused programmes estimated a 36% relative reduction in domestic-violence recidivism, though the evidence base draws largely on non-experimental designs (11). In the present study, effect sizes for domestic-violence offending were similar across follow-up periods.

These findings also have implications for the ethics of placebo-controlled trials in justice populations. A longstanding concern is that placebo assignment may deprive participants of active clinical benefit, an objection grounded in the principle of clinical equipoise (64, 65). In the ReINVEST context, this concern was mitigated because no pharmacotherapy for impulsive aggression had established efficacy against violent reoffending at the time of trial design (25). The present study concerns a question separate from this pharmacological equipoise, namely whether the structured psychiatric and psychosocial contact delivered during placebo participation was itself associated with reoffending outcomes. As the present results suggest, placebo assignment was not equivalent to the absence of clinical input. Participants randomized to placebo received psychiatric assessment, clinical monitoring, crisis support, and referral to external services. The ethical implication is that placebo conditions in justice-involved trials may embed a more sustained clinical environment than is often acknowledged, and that differences in the continuity and intensity of that environment may matter even when participants who dropped out are not wholly unsupported. Ethical evaluation of placebo-controlled trials in these populations should consider the broader clinical content of both arms rather than focusing exclusively on whether an active drug is withheld.

The pattern of non-progression itself points to the most plausible confounders. Men who reached randomization had, by definition, tolerated open-label sertraline, kept scheduled appointments through the run-in, and remained in contact with the trial team, whereas most non-randomized men left through lost contact, lost interest, or offence-related pathways. Completion of the run-in is a behavioral marker of motivation, medication adherence, and residential and social stability, and each of these attributes plausibly predicts lower reoffending in the absence of any effect of trial participation. The baseline covariates index criminal history and clinical severity but capture none of these attributes directly, so the estimates carry a risk of residual confounding that adjustment cannot remove. The E-value analyses bound the strength of unmeasured confounding required to explain away the point estimates and cannot demonstrate its absence.

### Strengths

This analysis has several methodological strengths. The counterfactual was informed by men drawn from the same trial pathway who completed the baseline assessment and entered the single-blind run-in phase but did not proceed to randomization, providing a closer comparison than an external control group. Both groups met the same eligibility criteria, were screened for major mental illness and clinically significant comorbidities, and completed the same baseline assessment battery capturing demographic, clinical, and criminal-history characteristics. Offending outcomes were ascertained from linked administrative records rather than self-report, reducing the risk of differential recall and social desirability bias. The estimand was prespecified and defined with respect to the baseline characteristics of men who entered the placebo arm.

The three assumptions required for causal interpretation were each addressed. The measured-covariate implication of conditional exchangeability was assessable and was examined through covariate balance, with standardized mean differences reduced after common-support restriction and to negligible levels after entropy balancing. Positivity was assessed empirically through the common-support restriction, which confined inference to the region of overlapping covariate profiles and reduced reliance on extrapolation. Consistency is supported at the level of the participation event, entry into the randomized phase under placebo, which is discrete and well specified. Documented clinical content during this phase comprised psychiatric assessment, nursing consultations, crisis support, and referral to external services, though the intensity and mix of this contact varied across individuals. This variation does not affect consistency, since the treatment is defined by entry into placebo participation rather than by the specific clinical contact received. The similarity of estimates under common-support restriction and entropy balancing indicated that the findings were not dependent on a single analysis strategy.

E-value analyses indicated that an unmeasured confounder would need rate-ratio associations with both participation and offending of 2.15 to 2.57 for violent offending and 3.01 to 3.38 for domestic-violence offending to explain away the point estimates. The domestic-violence estimates are more robust to a single unmeasured confounder than the violent-offending estimates, at both the point estimate and the confidence limit. A confounder capable of explaining away the domestic-violence associations would need to be associated with both participation and offending more strongly than the criminal-history and clinical covariates already included in the model, and confounders of this strength that are unrelated to prior offending, psychiatric history, and baseline clinical severity are difficult to specify. E-values quantify the minimum strength of unmeasured confounding required to explain away an estimate and cannot demonstrate that residual confounding is absent.

The external referral analyses further strengthened interpretation by addressing whether differences in earlier service engagement explained entry into placebo participation and by showing that the comparison group was not wholly unsupported after randomization. That adjustment for pre-randomization external referrals did not materially change the estimates, and that some post-randomization external referral activity was also observed in the comparison group, makes the observed association more conservative than a comparison between a supported group and an unsupported one.

### Limitations

The principal limitation is the potential for selection bias arising from non-random progression to randomization. The non-participation outcome for placebo participants was counterfactual and had to be estimated under conditional exchangeability. Men who entered the run-in but did not proceed to randomization informed this counterfactual outcome. Non-progression to randomization occurred for heterogeneous reasons, including loss to contact, loss of interest, incarceration during the run-in, and medical or psychiatric exclusion. These pathways do not make the analysis an observed comparison between randomized and non-randomized men, but they identify points at which unmeasured factors related to trial progression could also be related to later offending. To the extent that such factors were not captured by the baseline covariates, conditional exchangeability may not hold and the estimated participation effect may be biased. This concern is reduced, but not removed, by the shared eligibility criteria, common baseline assessment, and substantial covariate overlap under common support, which make the run-in non-randomized group a more credible source for estimating the counterfactual non-participation outcome than an external comparison group.

The referral analyses were also limited by measurement issues. External referral categories captured only services outside the trial, were not mutually exclusive, and were sparse in some categories. They did not capture the structured psychiatric contact delivered directly by the trial team, which was the core component of the participation exposure. Accordingly, these analyses could narrow one measurable pathway but could neither identify the mechanism nor exclude pathways operating through the internal clinical structure of trial participation. The effect-modification analyses were secondary, limited to the 12-month horizon, and not powered as primary tests of heterogeneity. Although continuous modifiers were modelled flexibly, the resulting estimates remained imprecise and should be interpreted cautiously.

The intensity of clinical contact varied across participants, and placebo participation was a complex, multi-component exposure. The estimated effect averages over the versions of participation delivered in this trial, does not identify which components carried the association, and may not transfer to trials or services delivering a different intensity or mix of clinical contact.

Offending was ascertained using administrative records, which capture matters that come to official attention. This may lead to under-ascertainment of the true rate of offending, which is especially relevant to domestic-violence offending. Whether underreporting differed between participation states cannot be determined, so the direction of any resulting bias is uncertain.

The study population comprised men, as a consequence of the design, because the ReINVEST cohort comprised men only. The findings do not extend to women, in whom the serotonin and impulsivity mechanisms targeted by the trial also operate but were not recruited. Inference is further limited to men with the clinical and offending profile required for trial entry, high trait impulsivity and repeat violent offending, and does not extend to perpetrators with less pronounced profiles or to populations outside the criminal justice system.

## Conclusion

In the ReINVEST clinical trial, placebo participation was associated with lower subsequent violent and domestic-violence offending than non-participation. The association was evident at both 12 and 24 months, was not explained by differences in external referral activity before randomization and was not accounted for by external referrals recorded after randomization. Although these analyses do not identify the mechanism, they are consistent with the view that the structured psychiatric contact and psychosocial support embedded in placebo participation constituted a clinically meaningful part of the trial condition. Because participation was not randomized, residual confounding by unmeasured factors cannot be excluded, although the E-value analyses indicated that any such confounder capable of explaining away the domestic-violence associations would need to be strongly associated with both participation and offending, beyond the measured covariates. If confirmed in prospective studies in which the components of clinical contact are randomized or systematically varied, the findings would support embedding structured psychiatric monitoring and psychosocial support in services for justice-involved men. These findings underscore the importance of characterizing the content of placebo participation when designing, reporting, and interpreting placebo-controlled trials in justice-involved populations.

## Data Availability

The individual participant data supporting the conclusions of this article are not publicly available because of conditions in the participant consent forms relating to the sensitive nature of the data and the terms of the data-linkage approvals. De-identified data may be made available by the corresponding author upon reasonable request and subject to relevant ethics and data custodian approvals.
