# Supplementary material for: Structured psychiatric care and psychosocial support during placebo participation: association with violent and domestic-violence offending in the ReINVEST trial
Source: Front Psychiatry. 2026 Aug 12;17:1843657. doi: 10.3389/fpsyt.2026.1843657 (PMC13506906; doi:10.3389/fpsyt.2026.1843657)
Supplement: Supplementary file 1 [file DataSheet1.pdf]

Structured psychiatric care and psychosocial support during placebo participation: association with violent and domestic-violence offending in the ReINVEST trial

Supplementary Table 1. Participant-level external referrals in placebo and non-randomised groups before and after randomisation/index date

Referral category	Placebo<br><br>total<br>n (%)	Placebo before randomization<br><br>n (%)	Placebo after randomization<br><br>n (%)	Entered run-in, not randomised<br><br>total<br>n (%)	Entered run-in, not randomized, before index date<br>n (%)	Entered run-in, not randomized, after index date<br>n (%)
Court assistance/support	134 (43.4)	39 (12.6)	108 (35.0)	57 (34.3)	40 (24.1)	28 (16.9)
Medical services (non-mental health)	53 (17.2)	10 (3.2)	47 (15.2)	20 (12.0)	12 (7.2)	10 (6.0)
Mental health services	30 (9.7)	6 (1.9)	25 (8.1)	15 (9.0)	9 (5.4)	6 (3.6)
Legal services	22 (7.1)	5 (1.6)	17 (5.5)	7 (4.2)	4 (2.4)	3 (1.8)
Police	5 (1.6)	2 (0.6)	3 (1.0)	2 (1.2)	1 (0.6)	1 (0.6)
Substance abuse/addiction programs	5 (1.6)	2 (0.6)	4 (1.3)	0 (0.0)	0 (0.0)	0 (0.0)
Charity organisations	1 (0.3)	0 (0.0)	1 (0.3)	1 (0.6)	0 (0.0)	1 (0.6)
Department of Communities & Justice	1 (0.3)	1 (0.3)	0 (0.0)	0 (0.0)	0 (0.0)	0 (0.0)
Disability services	1 (0.3)	0 (0.0)	1 (0.3)	0 (0.0)	0 (0.0)	0 (0.0)
Local community organisations/services	1 (0.3)	0 (0.0)	1 (0.3)	2 (1.2)	0 (0.0)	2 (1.2)
Victim support services	1 (0.3)	0 (0.0)	1 (0.3)	0 (0.0)	0 (0.0)	0 (0.0)
Employment assistance	0 (0.0)	0 (0.0)	0 (0.0)	1 (0.6)	1 (0.6)	0 (0.0)
Aboriginal health/support services	0 (0.0)	0 (0.0)	0 (0.0)	0 (0.0)	0 (0.0)	0 (0.0)
Emergency services (non-medical)	0 (0.0)	0 (0.0)	0 (0.0)	0 (0.0)	0 (0.0)	0 (0.0)
Housing support	0 (0.0)	0 (0.0)	0 (0.0)	0 (0.0)	0 (0.0)	0 (0.0)

The index date was defined as the screening date plus four weeks.

Supplementary Table 2. Prior external referrals and later placebo participation

Prior referral variable	Risk ratio (95% CI)	P-value
Court assistance/support	0.73 [0.59, 0.91]	0.004
Medical services (non-mental health)	0.70 [0.45, 1.09]	0.113
Mental health services	0.66 [0.37, 1.19]	0.171
Legal services	0.81 [0.45, 1.46]	0.488
Any external referral	0.73 [0.60, 0.89]	0.002

Risk ratios are from models predicting later placebo participation from pre-randomisation referral variables. Risk ratios below 1.0 indicate lower likelihood of later placebo participation among those with the corresponding prior referral.

Supplementary Table 3. Association between placebo participation and later offending before and after adjustment for prior external referrals

Outcome	Common support<br>Estimate (95% CI)	Entropy balanced<br>Estimate (95% CI)
Violent offending, 12 months	-0.20 [-0.43, -0.04]	-0.25 [-0.59, -0.02]
Violent offending, 24 months	-0.20 [-0.48, 0.01]	-0.26 [-0.65, 0.02]
Domestic-violence offending, 12 months	-0.41 [-0.67, -0.11]	-0.37 [-0.72, -0.07]
Domestic-violence offending, 24 months	-0.49 [-0.77, -0.13]	-0.45 [-0.81, -0.13]

Estimates are standardised mean count differences. Negative values indicate fewer offending events among placebo participants. Prior-adjusted models added pre-randomisation external referral variables to the adjustment set.

Supplementary Table 4. Interventional single-mediator analyses of post-randomisation external referrals for violent offending at 12 months

Mediator	Method	Indirect effect<br>(95% CI)	Direct effect<br>(95% CI)	Total effect<br>(95% CI)
Court assistance/support	Common support	0.02 [-0.01, 0.05]	-0.22 [-0.42, -0.09]	-0.20 [-0.42, -0.08]
Court assistance/support	Entropy balanced	0.02 [-0.01, 0.05]	-0.27 [-0.53, -0.10]	-0.26 [-0.51, -0.07]
Medical services (non-mental health)	Common support	0.00 [-0.01, 0.02]	-0.20 [-0.41, -0.07]	-0.20 [-0.41, -0.07]
Medical services (non-mental health)	Entropy balanced	0.00 [-0.02, 0.02]	-0.25 [-0.51, -0.07]	-0.25 [-0.51, -0.07]
Mental health services	Common support	0.00 [-0.01, 0.02]	-0.20 [-0.41, -0.07]	-0.20 [-0.41, -0.07]
Mental health services	Entropy balanced	0.00 [-0.01, 0.02]	-0.25 [-0.52, -0.07]	-0.25 [-0.51, -0.06]
Legal services	Common support	0.01 [-0.00, 0.03]	-0.21 [-0.41, -0.08]	-0.20 [-0.41, -0.07]
Legal services	Entropy balanced	0.01 [-0.00, 0.04]	-0.26 [-0.53, -0.08]	-0.25 [-0.51, -0.07]
Event count	Common support	0.03 [0.01, 0.05]	-0.23 [-0.43, -0.10]	-0.20 [-0.42, -0.07]
Event count	Entropy balanced	0.03 [0.00, 0.05]	-0.29 [-0.54, -0.11]	-0.26 [-0.51, -0.08]
Distinct service categories	Common support	0.02 [-0.00, 0.06]	-0.22 [-0.42, -0.09]	-0.20 [-0.41, -0.06]
Distinct service categories	Entropy balanced	0.02 [-0.01, 0.06]	-0.27 [-0.52, -0.09]	-0.25 [-0.51, -0.06]

Estimates are standardised mean count differences from single-mediator interventional analyses. Indirect effects quantify the portion of the association carried through the specified post-randomisation external referral variable. Negative direct or total effects indicate fewer offending events among placebo participants.

Supplementary Table 5. Interventional single-mediator analyses of post-randomisation external referrals for violent offending at 24 months

Mediator	Method	Indirect effect (95% CI)	Direct effect (95% CI)	Total effect (95% CI)
Court assistance/support	Common support	0.02 [-0.01, 0.06]	-0.23 [-0.47, -0.03]	-0.20 [-0.44, -0.01]
Court assistance/support	Entropy balanced	0.02 [-0.02, 0.06]	-0.28 [-0.59, -0.03]	-0.27 [-0.58, -0.00]
Medical services (non-mental health)	Common support	0.00 [-0.02, 0.03]	-0.20 [-0.45, -0.01]	-0.20 [-0.45, -0.01]
Medical services (non-mental health)	Entropy balanced	0.00 [-0.02, 0.03]	-0.26 [-0.59, -0.00]	-0.26 [-0.60, -0.00]
Mental health services	Common support	0.01 [-0.01, 0.03]	-0.20 [-0.44, -0.01]	-0.20 [-0.44, -0.01]
Mental health services	Entropy balanced	0.01 [-0.01, 0.04]	-0.27 [-0.59, -0.01]	-0.26 [-0.59, 0.00]
Legal services	Common support	0.02 [-0.00, 0.08]	-0.23 [-0.45, -0.04]	-0.21 [-0.44, -0.02]
Legal services	Entropy balanced	0.02 [-0.00, 0.07]	-0.28 [-0.62, -0.04]	-0.27 [-0.60, -0.01]
Event count	Common support	0.04 [-0.00, 0.08]	-0.24 [-0.48, -0.05]	-0.21 [-0.45, -0.01]
Event count	Entropy balanced	0.04 [-0.01, 0.08]	-0.31 [-0.62, -0.06]	-0.27 [-0.60, -0.01]
Distinct service categories	Common support	0.03 [-0.02, 0.08]	-0.23 [-0.47, -0.04]	-0.20 [-0.45, -0.01]
Distinct service categories	Entropy balanced	0.03 [-0.02, 0.08]	-0.29 [-0.60, -0.05]	-0.26 [-0.58, 0.00]

Estimates are standardised mean count differences from single-mediator interventional analyses. Indirect effects quantify the portion of the association carried through the specified post-randomisation external referral variable. Negative direct or total effects indicate fewer offending events among placebo participants.

Supplementary Table 6. Interventional single-mediator analyses of post-randomisation external referrals for domestic-violence offending at 12 months

Mediator	Method	Indirect effect (95% CI)	Direct effect (95% CI)	Total effect (95% CI)
Court assistance/support	Common support	-0.00 [-0.04, 0.04]	-0.41 [-0.68, -0.13]	-0.41 [-0.70, -0.12]
Court assistance/support	Entropy balanced	-0.00 [-0.03, 0.04]	-0.37 [-0.71, -0.12]	-0.37 [-0.73, -0.13]
Medical services (non-mental health)	Common support	0.00 [-0.02, 0.02]	-0.42 [-0.71, -0.12]	-0.41 [-0.70, -0.12]
Medical services (non-mental health)	Entropy balanced	-0.00 [-0.02, 0.02]	-0.37 [-0.72, -0.12]	-0.37 [-0.75, -0.13]
Mental health services	Common support	0.01 [-0.00, 0.03]	-0.42 [-0.71, -0.12]	-0.41 [-0.71, -0.11]
Mental health services	Entropy balanced	0.01 [-0.01, 0.04]	-0.38 [-0.74, -0.12]	-0.37 [-0.73, -0.11]
Legal services	Common support	0.02 [-0.00, 0.06]	-0.45 [-0.75, -0.14]	-0.43 [-0.71, -0.13]
Legal services	Entropy balanced	0.02 [-0.00, 0.06]	-0.40 [-0.76, -0.13]	-0.38 [-0.74, -0.13]
Event count	Common support	0.03 [-0.01, 0.07]	-0.45 [-0.74, -0.17]	-0.42 [-0.69, -0.13]
Event count	Entropy balanced	0.03 [-0.00, 0.07]	-0.42 [-0.76, -0.16]	-0.38 [-0.74, -0.13]
Distinct service categories	Common support	0.02 [-0.02, 0.07]	-0.43 [-0.71, -0.15]	-0.41 [-0.70, -0.11]
Distinct service categories	Entropy balanced	0.03 [-0.02, 0.07]	-0.40 [-0.72, -0.14]	-0.37 [-0.74, -0.11]

Estimates are standardised mean count differences from single-mediator interventional analyses. Indirect effects quantify the portion of the association carried through the specified post-randomisation external referral variable. Negative direct or total effects indicate fewer offending events among placebo participants.

Supplementary Table 7. Interventional single-mediator analyses of post-randomisation external referrals for domestic-violence offending at 24 months

Mediator	Method	Indirect effect (95% CI)	Direct effect (95% CI)	Total effect (95% CI)
Court assistance/support	Common support	0.01 [-0.04, 0.07]	-0.50 [-0.81, -0.21]	-0.49 [-0.81, -0.20]
Court assistance/support	Entropy balanced	0.01 [-0.03, 0.06]	-0.46 [-0.84, -0.15]	-0.45 [-0.85, -0.14]
Medical services (non-mental health)	Common support	0.00 [-0.03, 0.03]	-0.49 [-0.80, -0.19]	-0.49 [-0.81, -0.19]
Medical services (non-mental health)	Entropy balanced	0.01 [-0.03, 0.03]	-0.46 [-0.86, -0.15]	-0.45 [-0.85, -0.13]
Mental health services	Common support	0.01 [-0.01, 0.04]	-0.50 [-0.82, -0.20]	-0.49 [-0.81, -0.18]
Mental health services	Entropy balanced	0.02 [-0.01, 0.05]	-0.47 [-0.86, -0.15]	-0.46 [-0.85, -0.13]
Legal services	Common support	0.02 [-0.00, 0.08]	-0.53 [-0.82, -0.21]	-0.50 [-0.81, -0.20]
Legal services	Entropy balanced	0.02 [-0.00, 0.07]	-0.49 [-0.87, -0.17]	-0.47 [-0.85, -0.15]
Event count	Common support	0.04 [-0.01, 0.10]	-0.53 [-0.82, -0.22]	-0.49 [-0.81, -0.19]
Event count	Entropy balanced	0.05 [-0.01, 0.10]	-0.52 [-0.88, -0.20]	-0.47 [-0.85, -0.15]
Distinct service categories	Common support	0.04 [-0.02, 0.09]	-0.52 [-0.81, -0.21]	-0.49 [-0.81, -0.17]
Distinct service categories	Entropy balanced	0.04 [-0.01, 0.10]	-0.50 [-0.87, -0.20]	-0.46 [-0.86, -0.14]

Estimates are standardised mean count differences from single-mediator interventional analyses. Indirect effects quantify the portion of the association carried through the specified post-randomisation external referral variable. Negative direct or total effects indicate fewer offending events among placebo participants.

Supplementary Figure 1. Distribution of estimated propensity scores in the placebo and comparison groups before and after common-support restriction

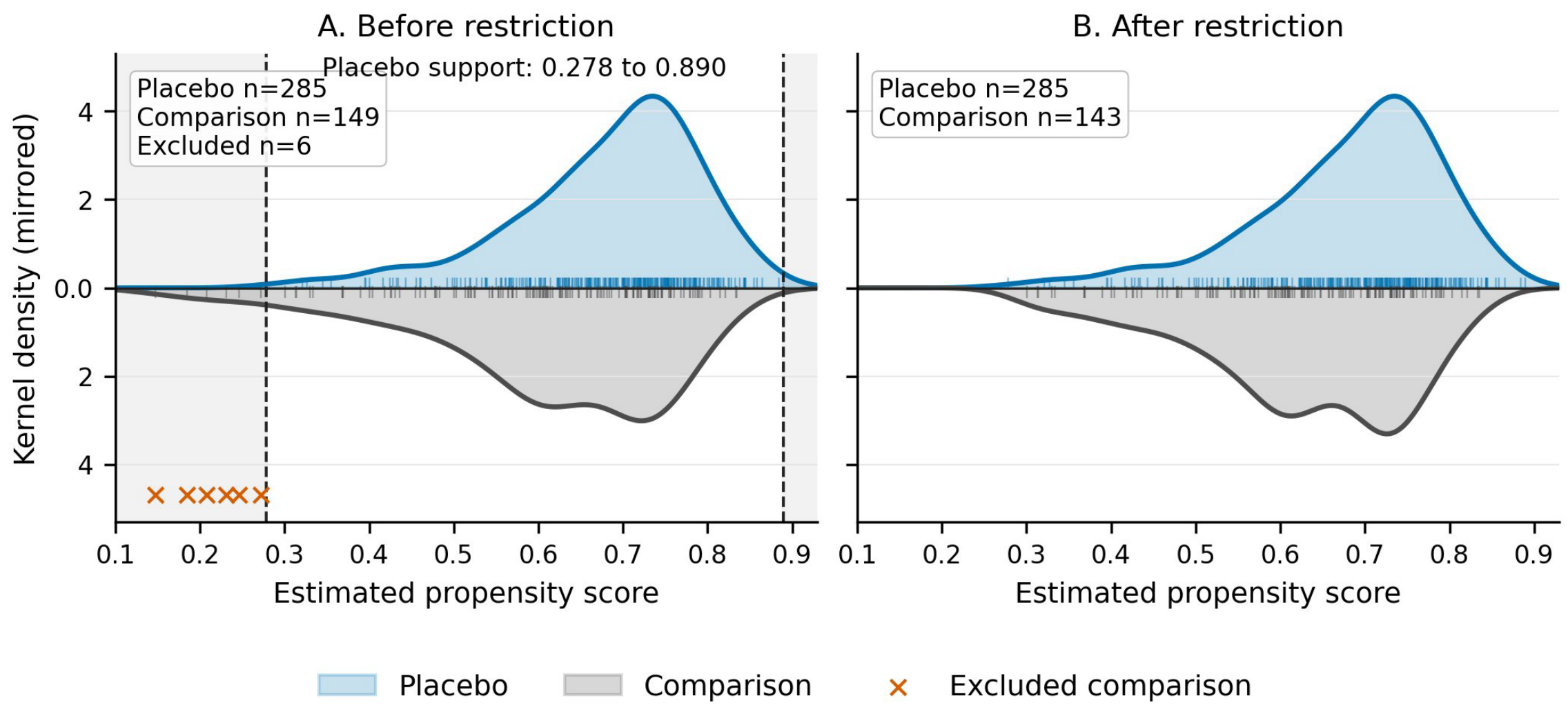

Propensity scores were estimated from a logistic regression of placebo participation on the baseline covariate set, fit on the complete-case analytic sample. The distributions overlapped substantially, and 6 of 149 comparison-group participants fell outside the range observed among placebo participants and were excluded by the common-support restriction.

Supplementary Table 8. Logistic regression model for placebo participation (propensity score model)

Covariate	Odds ratio (95% CI)	P-value
Indigenous	0.95 (0.60, 1.52)	0.841
Prior juvenile detention	0.60 (0.37, 0.97)	0.036
Any prior offence	0.95 (0.93, 0.98)	<0.001
Prior violent offence	1.05 (0.93, 1.18)	0.444
Prior DV offence	1.12 (1.02, 1.22)	0.013
Psychiatric history	0.93 (0.70, 1.25)	0.639
BDI score	1.02 (0.99, 1.06)	0.211
AIAQ total	1.00 (0.99, 1.01)	0.972
K10 score	1.00 (0.96, 1.04)	0.904
SF-12 PCS	1.00 (0.97, 1.02)	0.750
SF-12 MCS	1.02 (1.00, 1.05)	0.103

Odds ratios are from the logistic regression model used to estimate propensity scores for the common-support restriction, fit on the complete-case analytic sample of 434 men (285 placebo, 149 comparison). The model c-statistic was 0.65 (95% CI 0.59, 0.70).

Supplementary Table 9. Natural cubic spline specification for continuous effect modifiers

Effect modifier	25th	50th	75th
Prior violent offence count	1	2	3
Prior domestic-violence offence count	0	2	4
Depressive symptom severity	4.25	10.00	16.00
Anger and irritability	57.00	74.00	90.00
Psychological distress	9.00	14.00	22.00
Self-reported mental health functioning	30.10	41.65	50.71

Supplementary Table 10. Multiple-imputation sensitivity analysis of placebo participation and reoffending

Outcome	Estimator	Pooled ATT (95% CI)
Violent offending, 12 months	Common support	-0.17 (-0.33, -0.02)
Violent offending, 12 months	Entropy balanced	-0.16 (-0.28, -0.04)
Violent offending, 24 months	Common support	-0.19 (-0.39, 0.01)
Violent offending, 24 months	Entropy balanced	-0.18 (-0.34, -0.02)
Domestic-violence offending, 12 months	Common support	-0.38 (-0.65, -0.10)

Domestic-violence offending, 12 months	Entropy balanced	-0.35 (-0.56, -0.15)
Domestic-violence offending, 24 months	Common support	-0.47 (-0.79, -0.14)
Domestic-violence offending, 24 months	Entropy balanced	-0.44 (-0.69, -0.20)

Supplementary Table 11. Supplementary Table 11. Sensitivity analysis excluding men excluded after the run-in on medical or psychiatric grounds

Outcome	Estimator	ATT (95% CI)
Violent offending, 12 months	Common support	−0.24 (−0.45, −0.03)
Violent offending, 12 months	Entropy balanced	−0.26 (−0.49, −0.04)
Violent offending, 24 months	Common support	−0.25 (−0.48, 0.01)
Violent offending, 24 months	Entropy balanced	−0.27 (−0.55, −0.03)
Domestic-violence offending, 12 months	Common support	−0.50 (−0.85, −0.12)
Domestic-violence offending, 12 months	Entropy balanced	−0.44 (−0.86, −0.14)
Domestic-violence offending, 24 months	Common support	−0.59 (−1.00, −0.18)
Domestic-violence offending, 24 months	Entropy balanced	−0.56 (−1.02, −0.16)

Supplementary Table 12. E-value sensitivity analysis for unmeasured confounding

Outcome and follow-up	Rate ratio	E-value	Confidence-limit E-value
Violent offending, 12 months	0.63	2.57	1.19
Violent offending, 24 months	0.71	2.15	1.24
Domestic-violence offending, 12 months	0.50	3.38	1.93
Domestic-violence offending, 24 months	0.55	3.01	1.91

E-values were calculated from placebo-standardised rate ratios derived from the same g-computation predicted means used for the ATT analyses. Rate ratios below 1.00 were inverted before applying the E-value formula. Confidence-limit E-values used the confidence limit closest to the null. Higher E-values indicate that a stronger unmeasured confounder would be required to explain away the association.

Supplementary Table 13. Negative-binomial specification sensitivity analysis of placebo participation and reoffending

Outcome	Follow-up	Poisson ATT (95% CI)	Poisson dispersion ratio	Negative-binomial ATT (95% CI)
Violent offending	12 months	-0.19 (-0.38, -0.04)	1.40	-0.16 (-0.37, -0.02)
Violent offending	24 months	-0.22 (-0.51, -0.05)	1.45	-0.23 (-0.47, -0.03)
Domestic-violence offending	12 months	-0.37 (-0.81, -0.14)	2.04	-0.37 (-0.90, -0.12)
Domestic-violence offending	24 months	-0.49 (-0.92, -0.22)	2.13	-0.51 (-1.02, -0.20)

ATT denotes the average treatment effect on the treated, estimated by g-computation. Poisson dispersion ratios are Pearson chi-square statistics divided by residual degrees of freedom. Negative-binomial estimates refit the primary common-support outcome models under a negative-binomial specification.

Supplementary Table 14. Observed offending rates per 100 person-years in the community, by group and outcome domain

Outcome	Follow-up	Group	N	Events	Person-years	Observed rate per 100 person-years
Violent offending	12 months	Placebo	285	89	285.3	31.2
Violent offending	12 months	Comparison	149	81	148.7	54.5
Violent offending	24 months	Placebo	285	158	569.6	27.7
Violent offending	24 months	Comparison	149	131	297.5	44.0
Domestic-violence offending	12 months	Placebo	285	108	285.3	37.9
Domestic-violence offending	12 months	Comparison	149	144	148.7	96.9
Domestic-violence offending	24 months	Placebo	285	175	569.6	30.7
Domestic-violence offending	24 months	Comparison	149	202	297.5	67.9

Observed rates are the number of offending events per 100 person-years in the community during each follow-up window, in the complete-case analytic sample. Person-years reflect time at risk in the community over the 12- and 24-month windows.
