# Supplementary material for: Structured psychiatric care and psychosocial support during placebo participation: association with violent and domestic-violence offending in the ReINVEST trial
Source: Front Psychiatry. 2026 Aug 12;17:1843657. doi: 10.3389/fpsyt.2026.1843657 (PMC13506906; doi:10.3389/fpsyt.2026.1843657)
Supplement: Supplementary file 2 [file DataSheet2.pdf]

# Statistical Analysis Plan

## Placebo Trial Participation and Reoffending Outcomes in the ReINVEST Clinical Trial

**Analysis title:** Effect of placebo trial participation on violent and domestic-violence reoffending in the ReINVEST trial

**Study type:** Secondary analysis of a randomised, double-blind, placebo-controlled clinical trial

**Parent trial registration:** Australian New Zealand Clinical Trials Registry, ACTRN12613000442707

**Version:** 1.0

**Date:** 28th July 2025

---

## 1. Objectives

### 1.1 Primary objective

To estimate the placebo-standardised marginal difference in subsequent violent and domestic-violence reoffending associated with entry into the randomised trial structure under placebo, relative to not entering the randomised phase after completion of baseline and single-blind run-in procedures.

### 1.2 Secondary objective

To assess whether the estimated placebo participation effect varies across selected baseline clinical and criminal-history characteristics, including psychiatric history, prior violent offending, prior domestic-violence offending, depressive symptom severity, anger and irritability, psychological distress, and self-reported mental and physical health functioning.

### 1.3 Referral and support objective

To characterise the scope and frequency of clinician-delivered supports and external referrals recorded in the trial's clinical management system, and to examine whether external referrals recorded before randomisation explain selection into placebo participation or materially alter the estimated participation effect.

### 1.4 Exploratory pathway objective

To examine whether external referrals recorded after randomisation account for part of the estimated association between placebo participation and subsequent offending.

## 2. Study population

### 2.1 Source population

All men enrolled in the ReINVEST trial who completed the baseline assessment and entered the single-blind sertraline run-in phase.

### 2.2 Analytic groups

**Placebo group:** All participants randomised to placebo as part of the double-blind phase.

**Counterfactual-informing group:** Eligible men who completed baseline assessment and the single-blind run-in phase but did not proceed to randomisation. These men were not exposed to the subsequent structured clinical contact or repeated follow-up visits constituting the trial participation exposure.

### 2.3 Exclusions

Participants with non-positive person-time at risk in the community during a given follow-up window will be excluded from analyses using that window. The analytic sample size will be reported separately for each outcome and follow-up horizon.

## 3. Causal framework and estimand

### 3.1 Framework

The analysis adopts a potential-outcomes causal inference framework. The exposure of interest is placebo trial participation ($A = 1$) versus non-participation in the randomised phase ($A = 0$).

### 3.2 Primary estimand

The placebo-standardised average treatment effect on the treated (ATT):

$$\psi = E[Y(1) - Y(0) \mid A = 1]$$

where $Y(1)$ denotes the potential offending outcome under placebo participation and $Y(0)$ denotes the potential offending outcome under non-participation, averaged over the covariate distribution of the placebo group.

### 3.3 Identifying assumptions

Identification of the ATT requires three structural assumptions:

1. **Conditional exchangeability:** The probability of placebo participation versus non-participation is independent of potential outcomes given observed baseline covariates. Formally: $Y(a) \perp A \mid X$, for $a \in \{0, 1\}$.
2. **Positivity:** Every placebo participant has a non-zero probability of appearing in the counterfactual-informing group given their covariate profile. Formally: $P(A = 0 \mid X = x) > 0$ for all x in the support of $X \mid A = 1$.
3. **Consistency:** The observed outcome for each participant corresponds to the potential outcome under their actual exposure. Formally: $Y = Y(A)$.

## 4. Outcomes

### 4.1 Outcome domains

Two offending outcome domains will be assessed:

- **Violent offending:** Classified using Australian and New Zealand Standard Offence Classification categories 01 to 06.
- **Domestic-violence offending:** Identified using domestic-violence offence flags within the NSW Reoffending Database.

### 4.2 Outcome definitions

The outcome for each domain and horizon is the number of proven offence events occurring during the relevant follow-up window. Multiple offences recorded on the same date will be treated as a single event and classified according to the most serious offence recorded on that date.

### 4.3 Follow-up horizons

Outcomes will be analysed over two follow-up horizons from the screening date:

- 12 months (365 days)
- 24 months (730 days)

### 4.4 Person-time at risk

Person-time at risk in the community during each follow-up window will be incorporated as a log-transformed offset in all outcome models so that estimated differences reflect offending conditional on time at risk.

### 4.5 Outcome ascertainment

All offending outcomes will be ascertained by probabilistic linkage of trial records to the NSW Reoffending Database, which captures proven criminal offences from court and incarceration records.

## 5. Baseline covariates

Adjustment covariates are selected a priori as potential confounders of the participation-offending association. The covariate set comprises:

- Indigenous status (binary)
- History of juvenile detention (binary)
- Any prior offending (continuous count)
- Prior violent offending (continuous count)
- Prior domestic-violence offending (continuous count)
- Psychiatric history (binary)
- Baseline depressive symptom severity (Beck Depression Inventory, continuous)
- Baseline anger and irritability (Aggression-Impulsivity and Anger Questionnaire, continuous)
- Baseline psychological distress (Kessler-10, continuous)
- Baseline self-reported physical health functioning (SF-12 PCS, continuous)
- Baseline self-reported mental health functioning (SF-12 MCS, continuous)

The same baseline covariate set will be used in the primary propensity score and outcome models. Psychiatric history will be coded as a binary indicator. Analyses that specifically evaluate pre-randomisation referral history will additionally include indicators for prior court assistance/support, prior medical services other than mental health services, prior mental health services, prior legal services, and any prior external referral; these referral-augmented models will be reported separately from the primary baseline-covariate analysis.

## 6. Primary analysis (Phase 1): Total participation effect

### 6.1 Estimation procedure

The primary analysis estimates the placebo-standardised marginal difference in offending using a two-step procedure combining common-support restriction with g-computation.

**Step 1: Propensity score estimation and common-support restriction**

A logistic regression model will be fitted for the probability of belonging to the placebo group versus the counterfactual-informing group, with the baseline covariates specified in Section 5 as predictors. The analytic sample will then be restricted to participants whose estimated propensity score falls within the minimum-to-maximum range observed among placebo participants. This restriction confines inference to the region of shared covariate support and avoids extrapolation.

**Step 2: Outcome modelling and g-computation**

Within the common-support sample, the offending count outcome will be modelled using Poisson regression with an exposure indicator (A), the full baseline covariate set, and a log-transformed person-time offset. G-computation will be implemented by generating model-predicted outcomes for each placebo participant under two counterfactual scenarios:

- Participation as observed (A = 1), yielding $E[Y(1) \mid A = 1]$
- Non-participation (A = 0), with baseline covariates held at observed values, yielding $E[Y(0) \mid A = 1]$

The placebo-standardised participation effect will be computed as:

$\hat{\psi} = (1/n_1)\ \Sigma\ [\hat{Y}_i(1) - \hat{Y}_i(0)]$ for i: $A_i = 1$

**6.2 Diagnostics**

- Number of participants excluded by common-support restriction
- Range of propensity scores in each group before and after restriction
- Standardised mean differences on all baseline covariates before and after restriction

## 7. Sensitivity analysis for Phase 1: Entropy balancing within common support

A pre-specified sensitivity analysis will use entropy balancing as a second estimator for the same placebo-standardised ATT in the retained common-support population. Entropy-balancing weights will be constructed for comparison-group participants in the common-support sample to target first-moment balance on the baseline covariates specified in Section 5 among placebo participants. Weighted outcome models will be fitted using frequency weights in the same Poisson specification as the primary analysis, retaining the log-offset term. G-computation will be repeated over placebo participants using model predictions standardised to the placebo covariate distribution.

### 7.1 Diagnostics

Effective sample size of the reweighted comparison group, maximum weight value, and post-weighting standardised mean differences on all baseline covariates will be reported. Balance will be considered adequate when absolute standardised mean differences are below 0.10. Full-sample unweighted balance may be shown as a pre-restriction reference, but plausibility diagnostics for the ATT will be based on the common-support population.

## 8. Secondary analysis: effect modification

Effect-modification analyses will assess whether the placebo participation effect differs across selected baseline clinical and criminal-history characteristics. Psychiatric history will be examined as a binary modifier.

Continuous modifiers will include prior violent offence count, prior domestic-violence offence count, baseline depressive symptom severity, baseline anger and irritability, baseline psychological distress, baseline physical health functioning, and baseline mental health functioning.

Continuous modifiers will be modelled flexibly using natural cubic splines with three degrees of freedom, with knot positions placed at the empirical 25th, 50th, and 75th percentiles in the analytic sample.

Participation effects will be estimated at the 25th, 50th, and 75th percentiles of each continuous modifier, and the high-versus-low contrast will be defined as the difference between effects estimated at the 75th and 25th percentiles.

Interaction evidence will be assessed using Wald tests, and results will be interpreted cautiously as heterogeneity analyses rather than as primary efficacy tests.

## 9. Referral and support analyses

Structured clinician-contact data recorded in the trial's clinical management system will be summarised descriptively. Referral categories will be treated as non-mutually exclusive. The number of referral events, the number and percentage of participants with each referral category, and the distribution of referral categories will be reported by analytic group.

Referrals recorded before randomisation will be examined as potential sources of selection into placebo participation. These analyses will include models for placebo participation as a function of the pre-randomisation referral variables and sensitivity analyses repeating the primary participation-effect models after adding pre-randomisation referral indicators to the adjustment set.

Referrals recorded after randomisation will be examined as candidate intermediate variables that may account for part of the association between placebo participation and later offending. These analyses will estimate indirect, direct, and total components using single-mediator interventional analyses under the same common-support framework as the main analyses. Because external referrals do not capture the full structured clinical-contact package and were not independently assigned, these analyses will be interpreted as exploratory accounting analyses, not as definitive causal mediation analyses.

## 9. Missing data

A sensitivity analysis will address missing baseline covariate data under a missing-at-random assumption using multiple imputation by chained equations. The imputation model will include all baseline covariates, the exposure indicator, outcome counts, and log person-time at risk as predictors; exposure status, outcome counts, and time-at-risk variables will not themselves be imputed. Twenty imputed datasets will be generated. Within each imputed dataset, the common-support restriction will be re-applied and the Phase 1 common-support and entropy-balanced estimators will be re-estimated. Estimates will be pooled using Rubin's rules. This sensitivity analysis is restricted to the primary Phase 1 estimand.

## 10. Uncertainty estimation

Uncertainty will be quantified using nonparametric bootstrap resampling at the participant level with replacement. In each bootstrap replicate, the full estimation procedure will be repeated from propensity score fitting through g-computation. Percentile confidence intervals will be calculated from the empirical bootstrap distribution. The number of bootstrap replicates will be 1,000.

## 11. Descriptive analysis of clinician-delivered supports

Structured clinician-contact data recorded in the trial's clinical management system will be summarised descriptively. The total number of referral events, the mean number of referrals per participant, and the distribution of referrals across service categories will be reported. These data are intended as contextualising evidence for interpretation of any observed participation effect and are not subject to formal inferential analysis.

## 12. Outcome matrix

The following analyses will be conducted for each combination of outcome domain and follow-up horizon:

| Analysis | Outcome | Horizon | Estimator |
| --- | --- | --- | --- |
| Phase 1 primary | Violent offending count | 12 months | Common-support g-computation |
| Phase 1 primary | Violent offending count | 24 months | Common-support g-computation |
| Phase 1 primary | DV offending count | 12 months | Common-support g-computation |
| Phase 1 primary | DV offending count | 24 months | Common-support g-computation |
| Phase 1 sensitivity | Violent offending count | 12 months | Entropy-balanced g-computation within common support |
| Phase 1 sensitivity | Violent offending count | 24 months | Entropy-balanced g-computation within common support |
| Phase 1 sensitivity | DV offending count | 12 months | Entropy-balanced g-computation within common support |
| Phase 1 sensitivity | DV offending count | 24 months | Entropy-balanced g-computation within common support |
| Effect modification | Violent offending count | 12 months | Common-support g-computation with modifier interactions |
| Effect modification | DV offending count | 12 months | Common-support g-computation with modifier interactions |
| Referral selection/sensitivity | Violent and DV offending counts | 12 and 24 months | Referral-augmented common-support and entropy-balanced models |
| Referral accounting | Violent and DV offending counts | 12 and 24 months | Single-mediator interventional accounting analyses |

## 13. Software

All analyses will be conducted in Python. Data management will use pandas and NumPy. Propensity score estimation will use scikit-learn. Generalised linear models with offset terms and frequency weights will be fitted using statsmodels. Entropy-balancing optimisation will use SciPy. Multiple imputation will use scikit-learn iterative imputation. Parallelised bootstrapping will use joblib.

## 14. Amendments

Any amendments to this analysis plan made after the analysis has commenced will be documented, dated, and reported as deviations from the pre-specified plan.
